# Computational Analyses Reveal Deregulated Clock Genes Associated with Breast Cancer Development in Night Shift Workers

**DOI:** 10.3390/ijms25168659

**Published:** 2024-08-08

**Authors:** Silvia Vivarelli, Giovanna Spatari, Chiara Costa, Federica Giambò, Concettina Fenga

**Affiliations:** 1 Department of Biomedical and Dental Sciences, Morphological and Functional Imaging, Section of Occupational Medicine, University of Messina, 78712 Messina, Italy; giovanna.spatari@unime.it (G.S.); federicagiambo@gmail.com (F.G.); cfenga@unime.it (C.F.); 2 Department of Clinical and Experimental Medicine, University of Messina, 78712 Messina, Italy; chiara.costa@unime.it

**Keywords:** night shift work, breast cancer, clock genes, micro-RNAs, circadian rhythms, biomarkers, computational study

## Abstract

Breast cancer (BC) is the leading cause of cancer death among women worldwide. Women employed in shift jobs face heightened BC risk due to prolonged exposure to night shift work (NSW), classified as potentially carcinogenic by the International Agency for Research on Cancer (IARC). This risk is linked to disruptions in circadian rhythms governed by clock genes at the cellular level. However, the molecular mechanisms are unclear. This study aimed to assess clock genes as potential BC biomarkers among women exposed to long-term NSW. Clock gene expression was analysed in paired BC and normal breast tissues within Nurses’ Health Studies I and II GEO datasets. Validation was performed on additional gene expression datasets from healthy night shift workers and women with varying BC susceptibility, as well as single-cell sequencing datasets. Post-transcriptional regulators of clock genes were identified through miRNA analyses. Significant alterations in clock gene expression in BC compared to normal tissues were found. BHLHE40, CIART, CLOCK, PDPK1, and TIMELESS were over-expressed, while HLF, NFIL3, NPAS3, PER1, PER3, SIM1, and TEF were under-expressed. The downregulation of PER1 and TEF and upregulation of CLOCK correlated with increased BC risk in healthy women. Also, twenty-six miRNAs, including miR-10a, miR-21, miR-107, and miR-34, were identified as potential post-transcriptional regulators influenced by NSW. In conclusion, a panel of clock genes and circadian miRNAs are suggested as BC susceptibility biomarkers among night shift workers, supporting implications for risk stratification and early detection strategies.

## 1. Introduction

Breast cancer (BC) represents the most frequently diagnosed cancer among women worldwide. According to the Global Cancer Observatory, in 2022, 2.3 million women were diagnosed with BC, and 670,000 deaths have been counted globally [1]. In Europe, for the year 2024, it has been predicted that there will be the highest absolute number of deaths (89,300) in women [2]. Intriguingly, compared to less developed countries, in more developed ones there is a higher survival rate attributed to earlier diagnosis, better preventive measures, and improved access to treatment [3]. The aetiology of BC is multifaceted as it involves reproductive factors, genetic predisposition, family history, and lifestyle choices. Hormone-related factors such as early menarche, late menopause, delayed pregnancies, and lower childbirth numbers are linked to BC development. Also, women with a family history of BC or specific mutations such as BRCA1 and BRCA2 are at higher risk. In addition, multiple lifestyle factors, including low physical activity, alcohol consumption, smoking, and a high-fat diet, are known to elevate the risk of developing this tumour [4]. Likewise, several occupational exposures have been associated with an elevated risk of BC [5].

Notably, BC aetiology extends beyond this broad range of non-modifiable and modifiable risk factors, appearing to be tightly linked with circadian clock alterations as a significant contributor [6]. Circadian rhythms are pivotal for life, having the purpose of synchronizing our homeostatic processes with the rhythmic alternation of daytime and nighttime [7]. At the cellular level, they are governed by a conserved group of clock genes with established self-regulatory feedback loops, although the signal transduction pathways remain yet to be fully elucidated [8]. Clock gene alterations have been shown to significantly contribute to BC initiation, progression, and invasion, either by modifying neoplastic cell homeostasis or the tumour microenvironment [9,10]. Within the mammary epithelium, it has been proven that clock gene impairments (e.g., point mutations, alteration of transcription or translation, post-translational modifications) may lead to cell cycle deregulation, which can be correlated with tumour progression and more aggressive BC [11].

Several studies indicate a 30–50% increased BC risk in countries with high light at night (LAN) exposure compared to those with lower exposure [12]. Altered clock gene expression in BC may be caused by disrupted circadian rhythm of melatonin synthesis due to a permanent exposure to artificial LAN, which particularly relates to night shift work (NSW) [5,13,14]. In fact, it has been observed that women who are persistently exposed to NSW and, consequently, are highly exposed to LAN, such as nurses, have a higher frequency of developing BC. On these bases, since 2019, NSW has been considered probably carcinogenic to humans by the International Agency for Research on Cancer (IARC) [15]. One of the most extensive American studies, the Nurses’ Health Studies I and II (NHS I and II), assessed the risk of BC linked with NSW, confirming a heightened BC risk among workers engaged in prolonged night shifts [16,17]. This observation has been corroborated by a number of multicentric studies, confirming the existence of a positive association between NSW and BC risk, particularly in high-intensity and/or long-duration patterns [18,19].

**Figure 1:**
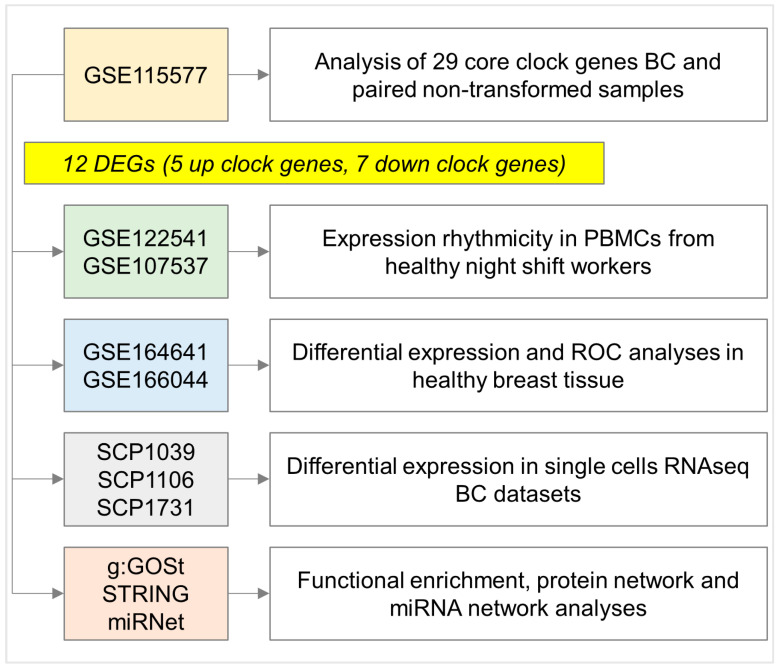
Flow chart of this study. DEGs, differentially expressed genes. GSE, gene set enrichment. PBMCs, peripheral blood mononuclear cells. ROC, receiver operating characteristic curve. BC, breast cancer. g: GOSt, functional enrichment from g: Profiler. STRING, protein–protein association networks. miRNet, miRNA network visual analytics platform.

From a mechanistical point of view, decreased nocturnal melatonin production may increase the pro-cancer effects of oestrogens, overall contributing to BC development [20]. Loss of expression of certain circadian clock genes has been found to be associated with BC progression in human studies [21]. Another hypothesized mechanism linking NSW to BC could be through the expression of some micro-RNAs (miRNAs) showing oscillations during the day [22]. Despite significant advances being made in the field of chronobiology, the molecular basis of malignant transformation in women chronically exposed to NSW is still debated. Hence, the objective of this study is to identify specific clock genes that could serve as promising biomarkers for BC while also exploring their association with varying susceptibility in women who work shifts at night. To achieve this goal, as summarized in Figure 1, a differential gene expression analysis of the clock gene candidate pool was performed using BC samples from the Gene Expression Omnibus (GEO) Dataset of NHS I and II.

These samples included nurses who developed BC and were exposed to NSW. The differential expression of clock genes was validated in multiple gene expression datasets from whole blood samples of healthy night shift workers, including female nurses, and BC tissue samples from healthy women with diverse susceptibilities to BC development. Furthermore, this analysis comprised the cell-specific expression patterns of the selected clock genes in both non-cancerous and cancerous breast tissues, along with their functional pathways and upstream epigenetic regulation.

## 2. Results

### 2.1. Differential Expression Analyses in NHS Dataset Highlights a Panel of Deregulated Clock Genes in BC Samples Compared with Matching Non-Transformed Breast Tissues

To assess the effect of NSW on BC development, a panel of 29 clock genes and 9 BC-related genes were strategically selected (Supplementary Table S1). Differential gene expression analyses were conducted in 623 BC and paired adjacent normal (Adj-N) breast samples from NHS I and II. Population statistics are reported in Table 1.

**Figure 2:**
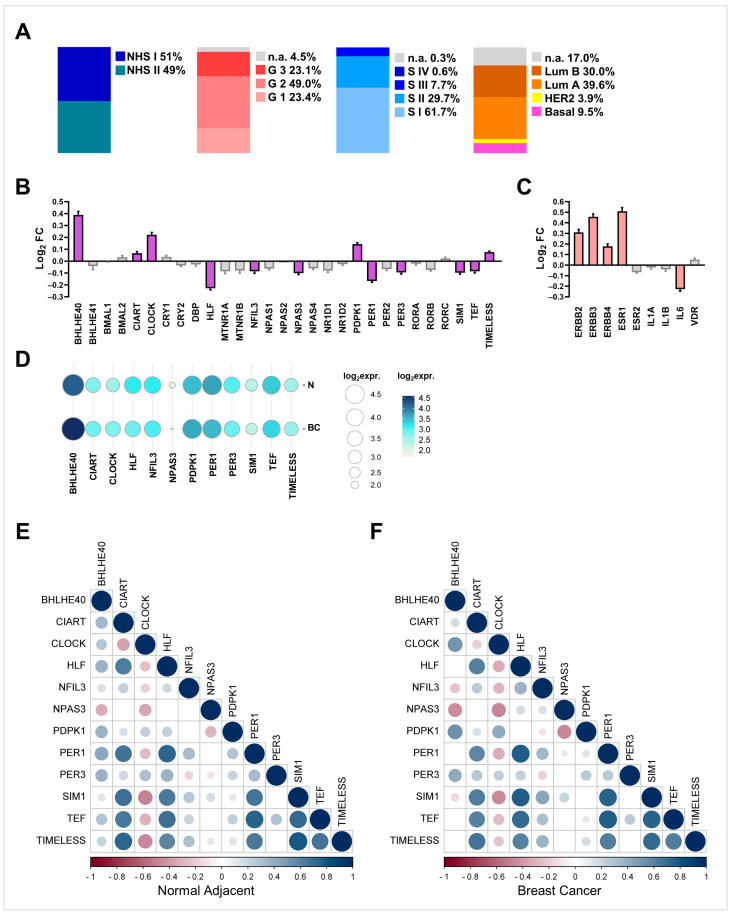
NHS cohort statistics and significantly deregulated clock genes and BC genes in GSE115577. (**A**) Stacked bar graphs illustrating percentage frequencies for the following stratifications: NHS cohorts I vs. II, grade, stage, and IHC BC types; (**B**) differential expressions of analysed core clock genes expressed as fold differences (Log2FC). Purple colour bars indicate significant values, grey bars not significant; (**C**) differential expressions of pivotal BC genes expressed as fold differences (Log2FC). Pink colour bars indicate significant values, grey bars not significant; (**D**) relative expression of significantly deregulated clock genes. The size and colour of the circles indicate the expression level (mean log2 expression value); (**E**,**F**) correlograms of Spearman correlations between deregulated core clock genes in adjacent non-cancerous breast tissue (E, *n* = 623) and BC tissue (F, *n* = 623). Blue colour indicates positive correlations (rho values > 1), while red colour indicates negative correlations (rho values < 1). Only significant correlations with *p* value ≥ 0.05 are reported.

Overall, the majority of BC cases were Luminal A, grade 2, and stage I (Figure 2A). The analysis revealed that 12 clock genes were significantly deregulated in both NHS I and NHS II (Figure 2B, Supplementary Figure S1). In particular, BHLHE40, CIART, CLOCK, PDPK1, and TIMELESS were significantly over-expressed, whereas HLF, NFIL3, NPAS3, PER1, PER3, SIM1, and TEF were significantly down-expressed (Figure 2B).

Among the differentially expressed genes, BHLHE40 was relatively highly expressed, while NPAS3 was expressed to a relatively low extent (Figure 2D). Concerning BC genes, while ERBB2, ERBB3, and ERBB4 were significantly upregulated in the BC samples compared with Adj-N, IL6 was significantly downregulated (Figure 2C). Interestingly, IL6 is involved not only in BC immune modulation but also in clock-controlled metabolism (Supplementary Figure S2). Additionally, the correlation analyses exhibited comparable trends of rho values both in the Adj-N and BC samples. However, while in the Adj-N samples, BHLHE40 was positively correlated with both NFIL3 and SIM1 (rho = 0.117, *p* = 0.003; rho = 0.124, *p* = 0.002, respectively), in the BC samples, they were negatively correlated (rho = −0.237, *p* < 0.0001; rho = −0.131, *p* = 0.001, respectively; Figure 2E,F).

**Figure 3:**
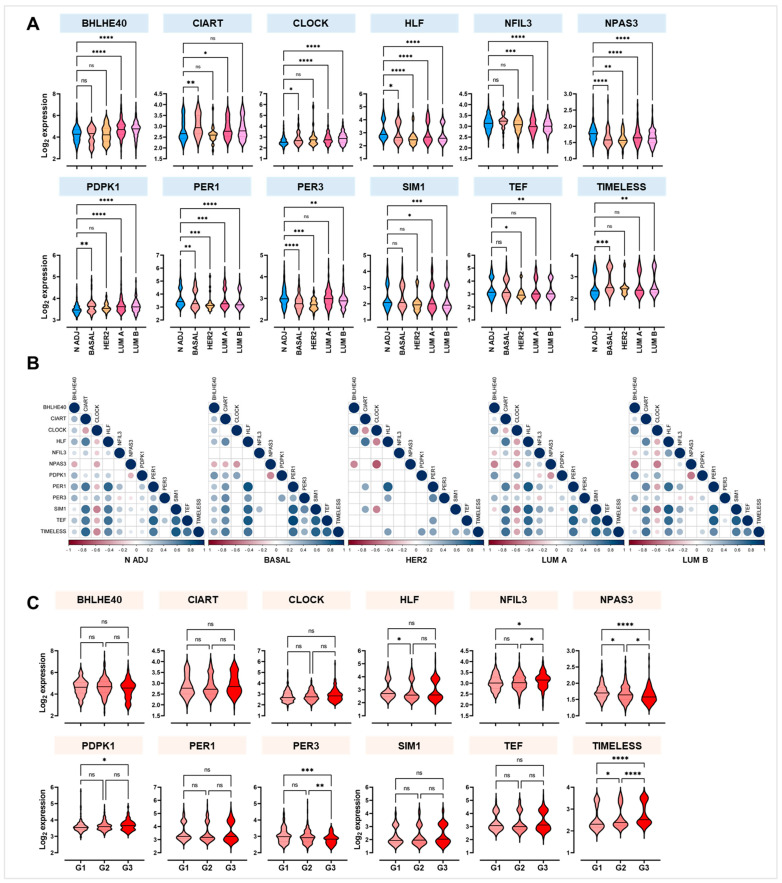
Stratification of significantly deregulated clock genes in GSE115577 based on major molecular types and tumour grade. (**A**) Violin plots with median of 12 significantly deregulated clock genes (only paired cancerous and non-cancerous samples with known IHC phenotypes were considered in this analysis) based on BC molecular subtype. Samples are divided into normal adjacent (N ADJ, *n* = 517), basal (*n* = 59), HER2-positive (HER2, *n* = 24), Luminal A (LUM A, *n* = 247), and Luminal B (LUM B, *n* = 187). (**B**) Correlograms of Spearman correlations between deregulated core clock genes in N ADJ, BASAL, HER2, LUM A, and LUM B (from left to right). Blue colour indicates positive correlations (rho values > 1), while red colour indicates negative correlations (rho values < 1). Only significant correlations with *p* value ≥ 0.05 are reported. (**C**) Violin plots with median of 12 significantly deregulated clock genes in BC samples stratified based on their grade from 1 to 3 (G1, *n* = 146; G2, *n* = 305; G3, *n* = 144). * *p* < 0.05; ** *p* < 0.01; *** *p* < 0.001; **** *p* < 0.0001; ns = not significant.

Furthermore, the BC samples were stratified based on IHC tumour subtype [23]. HLF, NPAS3, and PER1 were significantly downregulated within all four BC subgroups. With the exception of CIART, all clock genes were significantly deregulated in the Lum B samples compared to Adj-N (Figure 3A).

Moreover, the correlation matrices showed an overlapping distribution of rho values. In contrast with Adj-N, in the Lum A and Lum B samples, BHLHE40 was negatively correlated with both NFIL3 (rho = −0.247, *p* < 0.0001; rho = −0.223, *p* = 0.002, respectively) and SIM1 (rho = −0.183, *p* = 0.004; rho = −0.204, *p* = 0.005, respectively; Figure 3B).

Clock gene expression was evaluated in BC samples when stratified based on their grade. As shown in Figure 3C, NPAS3 and PER3 were significantly downregulated in both the G2 and G3 samples compared with G1. Meanwhile, PDPK1 and TIMELESS were significantly upregulated. Strikingly, no difference was found in clock gene expression when the samples were stratified based on BC stage (Supplementary Figure S3). Also, survival analyses and log-rank tests demonstrated that both RFS and DRFS were comparable in women with low vs. high clock gene expression (Supplementary Figure S4).

### 2.2. Clock Gene Expression in PBMCs from Healthy Night Shift Workers Does Not Show Differential Rhythmicity Compared with Day Shift Workers

To assess the effect of NSW on the expression of clock genes within healthy workers, two different gene expression datasets were analysed. In both studies, the human peripheral blood mononuclear cell (PBMC) transcriptome was considered. In GSE122541, total RNA was obtained from PBMCs isolated from blood every three hours in 3 night shift and 3 day shift nurses on a day off subsequent to repeated night or day rotation schedule. Meanwhile, in GSE107537, total RNA was obtained from PBMCs isolated from blood every four hours during a simulated day or night shift. Interestingly, none of the 12 clock genes analysed showed significantly different rhythmicity during NSW (Supplementary Figure S5).

### 2.3. PER1, TEF, and CLOCK Genes as Novel Putative Biomarkers of Breast Cancer Susceptibility

**Figure 4:**
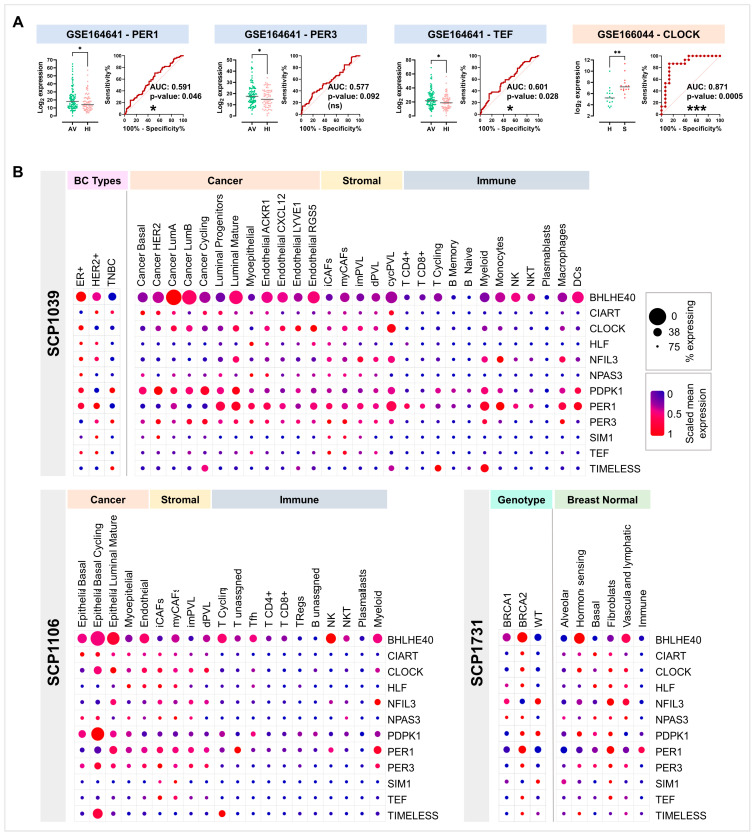
Clock gene expression in BC-susceptible subjects and single-cell RNA datasets of cancerous and non-cancerous human breast tissues. (**A**) Gene expression of significantly deregulated clock transcripts in breast tissue from healthy women with different degrees of risk of developing BC (GSE164641) or from healthy women that never developed BC or developed it years later (GSE166044). Dot plots with median log_2_ expression (left) and ROC analysis with AUC (right). AV, average risk of BC; HI, high risk of BC. H, healthy; S, susceptible. * *p* < 0.05; ** *p* < 0.01. (**B**) Selected clock gene expression in single-cell RNA datasets: dot plots with relative gene expression. The diameter of the dots is proportional to the number of cells expressing the gene, and the colour of the dots is proportional to the scaled mean expression (from blue to red). SCP1039 (100,064 cells from primary untreated BC biopsies), SCP1106 (24,271 cells from triple-negative BC biopsies), and SCP1731 (52,681 cells from healthy breast biopsies). * *p* < 0.05; *** *p* < 0.001; ns = not significant.

In order to evaluate the expression of the identified clock genes in breast biopsies from healthy women with different BC risks, two different gene expression datasets were considered. Firstly, GSE164641 was considered, which includes breast tissue samples from 162 women at average (91) or high (71) risk to develop BC, according to the Tyrer–Cuzick model [24]. Interestingly, PER1, PER3 and TEF displayed a significantly lower expression in high-risk samples compared with average-risk ones. The ROC curves a showed significant AUC for PER1 (0.591, *p* = 0.046) and TEF (0.601, *p* = 0.026; Figure 4A). These significant AUC performances suggest that both PER1 and TEF expression levels can be considered predictive discriminators between healthy women with a high vs. average risk of developing BC.

Additionally, in GSE166044, total RNA was extracted from healthy breast biopsies from women that either remained healthy (healthy group, *n* = 15) or were diagnosed with breast cancer a few years post-donation (susceptible group, *n* = 15). Differential expression analyses demonstrated that CLOCK was significantly overexpressed in breast samples from susceptible women compared with the healthy controls (Figure 4A). In line with this result, the ROC analysis showed a high and significant AUC (0.871, *p* = 0.0005), suggesting that CLOCK expression assessment may be used as a predictive marker of BC susceptibility.

### 2.4. Core Clock Gene Analyses in Single-Cell RNA-Seq Datasets Highlight Conserved Tissue-Specific Pattern of Expression

In order to assess whether the clock genes were differentially expressed depending on the nature of the tissue, three different single-cell RNA-seq experiments were examined. As shown in Figure 4B, in SCP1039 (100,064 cells from primary untreated BC), each clock gene presented a selected pattern of scaled mean expression as well as a percentage expression, depending on the tumour type. For instance, CLOCK was selectively expressed in ER+ cancer, whereas PER1 and TEF were highly expressed in both ER+ and HER+, and finally PER3 was expressed in HER2+ and TNBC. When cells from the same dataset were stratified based on cellular type, CLOCK and PER3 were preferentially expressed in tumour and stomal cells, PER1 was selectively expressed in immune cells and stromal cells but not in cancer cells, and TEF was selectively expressed in CAF subtypes.

Moreover, within SCP1106 (24,271 cells from TN BC), the same pattern of expression was confirmed for CLOCK and PER3; also, PER1 was selectively expressed in only a few immune subtypes (i.e., T cells and myeloid cells), and TEF was selectively expressed in CAFs (Figure 4B).

Finally, within SCP1731 (52,681 epithelial and stromal healthy breast cells), CLOCK, PER1, PER3, and TEF were selectively expressed in BRCA2-mutated cells. Also, while almost all genes were expressed in fibroblasts, only PER1 was expressed in immune cells, whereas both CLOCK and PER1 were expressed in hormone-sensing cells. Finally, only NPAS3 was expressed in alveolar cells (Figure 4B). Altogether, single-cell gene expression analyses suggest that every clock gene had a conserved cell-specific pattern of expression in both transformed and non-transformed human breast tissues.

### 2.5. Functional Enrichment and miRNA Network Analyses Reveal the Involvement of Pivotal Pathways and Post-Transcriptional Regulators in Transformed and Non-Transformed Breast Tissues

**Figure 5:**
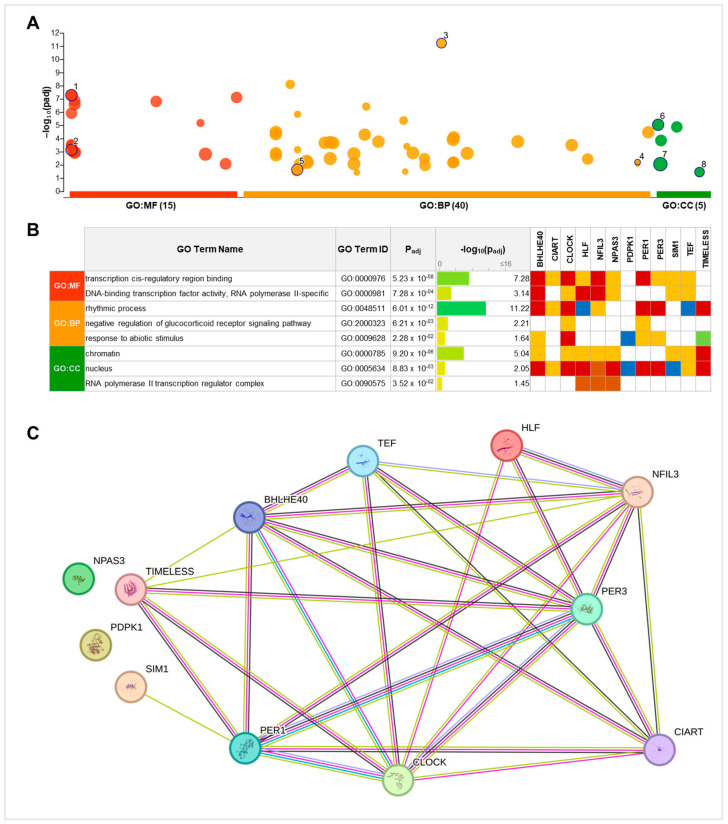
g: GOSt functional network analysis of differentially expressed clock genes in GSE115577. (**A**) g: GOSt multi-query Manhattan plot; *X*-axis shows the functional terms grouped and colour-coded by data source, and *Y*-axis shows the −log_10_ value of *p*-adjusted values. Highlighted driver GO terms are numbered from 1 to 8. (**B**) Table summarizing multi-query results with significant *p*-adjusted values and genes involved for 8 driver GO terms (square colours for different evidence; red: inferred from experiment, yellow: sequence or structural similarity, brown: genetic or physical interaction, blue: reviewed computational analysis, green: inferred by curator). MF, molecular function; BP, biological process; CC, cellular component; (**C**) STRING plot, with edges representing protein–protein associations (light green line indicates association through text mining, black line indicates interaction due to co-expression, light purple line indicates protein homology, deep purple line indicates known interaction experimentally determined, light blue line indicates known interaction from curated databases).

Through the g: GOSt tool (g: profiler), functional enrichment analysis was carried out for significantly deregulated clock genes. As shown in Figure 5A, a total of 15 GO molecular functions (MFs), 40 GO biological processes (BPs), and 5 GO cellular components (CCs) were significantly enriched (see Supplementary Table S2 for details). Among these, two MFs, three BPs, and three CCs included driver terms in GO (Figure 5B). Interestingly, in addition to the CC “nucleus”, five BPs and one MF exhibited an intersection size corresponding to all 12 clock genes (Supplementary Table S2).

Moreover, the STRING network analysis evidenced that protein products corresponding to 10 clock genes out of 12 were functionally interconnected, suggesting their concerted response not only to rhythmic regulatory processes but also to a number of metabolic pathways (Figure 5C).

**Figure 6:**
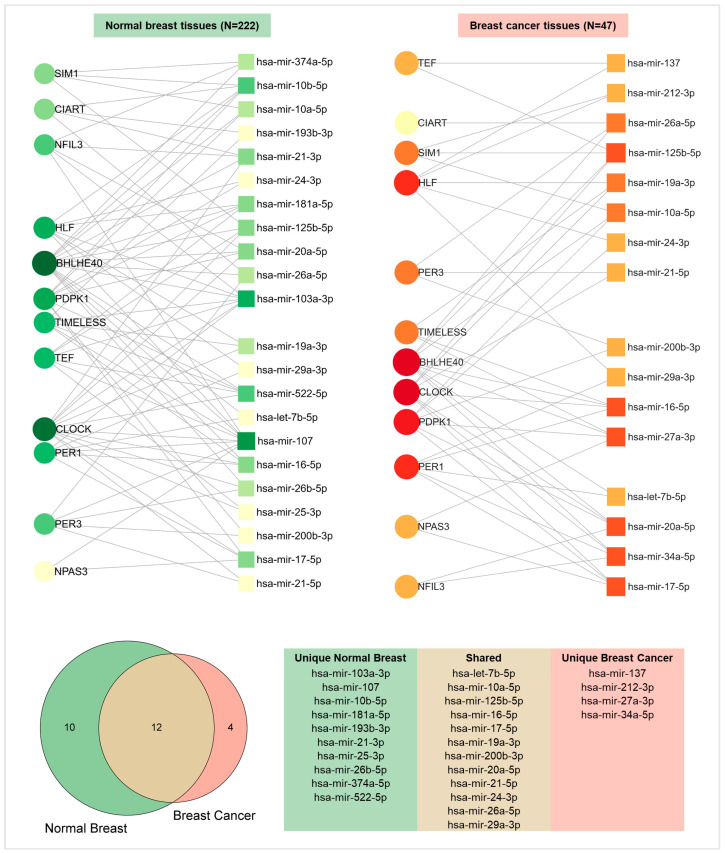
miRNet miRNA–target interaction network of differentially expressed clock genes in normal breast and breast cancer tissues. Upper left: network plot highlighting the interactions (grey nodes) between 12 clock genes and 22 miRNAs in normal breast samples. Upper right: network plot highlighting the interactions (grey nodes) between 12 clock genes and 16 miRNAs in BC samples. Lower left: Venn diagram showing intersections between miRNet queries. Lower right: list of miRNA post-transcriptional regulators of clock genes in three Venn subgroups (unique normal breast, shared, unique BC).

Finally, in order to identify which miRNAs might be putatively involved in the posttranscriptional regulation of clock genes, an miRNet analysis was carried out both in non-transformed and neoplastic breast tissues. The regulatory networks displayed in Figure 6 highlighted a total of 22 miRNAs in the normal samples and 16 miRNAs in the BC samples. Each miRNA modulated the expression of many clock genes. In particular, 12 miRNAs were found in both cancerous and non-cancerous tissues.

## 3. Discussion

Nurse Health Studies (NHSs) are among the largest prospective investigations concerning the risk factors associated with major chronic diseases in nurses working night shift. NSW was studied as a factor increasing BC risk [16,17,18,25]. The NHS gene expression dataset, which we investigated in our study, was previously employed to define BC molecular subtypes, their association with body mass index, adiposity, and inflammatory dietary patterns [26,27,28]. It includes a total of 1577 breast samples, including 623 paired cancerous and non-cancerous ones. We considered only paired samples to analyse the differential expression of the core clock genes (Table S1), known to orchestrate biological rhythms at the cellular level and, when deregulated, to contribute to BC development and progression [21,29].

Among the 29 clock genes analysed, we found that 12 were significantly deregulated in BC tissues compared with non-transformed paired ones, consistently in both NHS I and NHS II subgroups. In particular, while BHLHE40, CIART, CLOCK, PDPK1, and TIMELESS were significantly over-expressed, HLF, NFIL3, NPAS3, PER1, PER3, SIM1 and TEF were significantly down-expressed. Among these genes, NPAS3 and PER3 were highly downregulated in higher-grade BC compared with lower-grade BC, while PDPK1 and TIMELESS were significantly upregulated (Figure 3). In line with our findings, in a Polish study, it was observed that CLOCK and TIMELESS were over-expressed while PER1 and PER3 were down-expressed in BC compared to normal adjacent samples [11]. Additionally, two studies independently proved that a higher expression of TIMELESS and CLOCK and a lower expression of PER3 were associated with a higher BC grade, a lower metastasis-free survival rate, and an overall worse prognosis [21,30]. Finally, very recent research confirmed that in Lum A BC, compared to paired noncancerous tissue, PER1, PER2, HLF, TEF, and NFIL3 were downregulated, whereas CLOCK, CIART, and BHLHE40 were upregulated [31].

Intriguingly, while other groups found high PER2, PER3, and CRY2 expression and low TIMELESS expression were significantly associated with either longer metastasis-free survival or longer overall survival, in the NHS datasets, we could not find any correlation between clock genes’ expression levels and recurrence-free survival (Supplementary Figure S4). This difference might be explained by the variance in survival outcomes considered within the diverse studies [21,30].

Subsequently, in order to verify rhythmic clock gene expression in healthy workers, we analysed two different gene expression datasets from PBMC samples of day shift versus night shift workers. One of the datasets included nurses undertaking different rotation shifts. Notably, for none of the studies were we able to register any change in time-dependent fluctuations in clock gene expression (Supplementary Figure S5). This result could be explained by tissue-specific physiological and metabolic gene functions [32]. Our finding is in line with other studies, where PER1 or PER2 loses its normal day shift rhythmicity following NSW, while PER3 was significantly rhythmic in both shift conditions [33,34]. Also, Hattammaru et al. carried out a transcriptome analysis from beard-hair follicle cells and highlighted that, despite the observed rhythmic variation in PER3 in night shift workers, there was substantial variability between individuals [35].

In summary, the disruptive effects on clock gene rhythmicity seem to strictly depend on the nature of the tissue examined [36]. In light of that, a differential expression analysis was performed in single-cell RNA-seq datasets of cancerous and non-cancerous breast microenvironments, highlighting the selective expression of the clock genes within specific cellular components. For instance, CLOCK and PER3 were preferentially expressed in tumour and stomal cells, while PER1 was selectively expressed in immune cells and stromal cells but not in cancer cells. Finally, TEF was selectively expressed in CAFs (Figure 4B).

Given the above-described role of clock genes in the PBMCs of healthy workers, we opted to validate the predictive value of the same genes in two datasets of breast tissues from healthy women with diverse susceptibilities to develop BC. In one dataset with samples from women with different BC risks, PER1, PER3, and TEF downregulation was associated with higher risk, further confirming what was observed in the NHS datasets. Among these genes, both PER1 and TEF revealed predictive value, as demonstrated by their significant AUC performances. In the second dataset, we found greater CLOCK upregulation in breast tissue from healthy women that developed BC years later compared those that did not, with very high and significant AUC performance. Collectively, these results suggest that PER1, TEF, and CLOCK hold considerable promise in the early detection and risk stratification of BC, potentially guiding preventative strategies and personalized interventions for women at increased risk [37].

To further investigate the role of the identified clock genes, we performed a functional analysis, which evidenced that they regulate many processes, not only related to circadian rhythms and chronobiology but also to nuclear activity, transcription, and metabolism (Supplementary Table S2). Also, with the exclusion of NPAS3 and PDPK1, proteins coded by these genes are functionally linked, as proved by the STRING analysis. Overall, these findings underscore the multifaceted influence of the identified clock genes on several cellular functions and metabolic pathways, reinforcing their role as valuable biomarkers.

Regarding epigenetic regulation, the miRNet analysis highlighted a total of 26 miRNAs that might inhibit the expression of the 12 clock genes. Among them, 10 are specifically expressed in normal breast tissue, while only 4 are in BC. Interestingly, mir-10a, mir-21, mir-107, and mir-34 were previously found to be differentially methylated in night shift workers compared to day workers; hence, they might be suggested as putative circadian miRNAs [38]. This finding suggests an explorable role for these miRNAs as biomarkers for assessing BC risk in women chronically exposed to NSW. Moreover, it provides a new avenue for implementing novel research about the epigenetic mechanisms linking NSW to increased BC risk.

In conclusion, our computational study allowed for the identification of a panel of clock genes and circadian miRNAs which can be suggested as biomarkers of susceptibility, useful for identifying groups of night shift workers at higher risk of developing BC. In particular, PER1, TEF, and CLOCK may be recommended as predictive markers for BC susceptibility. This study has a number of strengths, but also limitations. One of the main strengths is the comprehensive analysis of clock gene expression across multiple datasets, including paired BC and normal tissues, as well as single-cell RNA-sequencing data. Also, leveraging large-scale datasets from NHS I and II adds robustness to our findings, given the extensive follow-up and detailed exposure assessment made in these cohorts over time.

However, there are also limitations. Firstly, the study relies on existing gene expression datasets; hence, while we identified significant associations between specific clock genes’ expression and BC risk, the cross-sectional nature of the data limits our ability to infer causality. Moreover, the lack of rhythmicity observed in clock genes from the PBMCs of healthy night shift workers highlights the need for tissue-specific investigations, as peripheral tissues may not accurately reflect changes occurring in the breast tissue. Furthermore, this study relies on pre-existing gene expression datasets, which limits the ability to control for potential confounding factors associated with such data. For example, variability in sample handling could introduce biases. Additionally, the absence of experimental validation across diverse tissue types constitutes a significant limitation. Future research should address this by employing experimental approaches such as tissue-specific in vitro assays or animal models to confirm the findings and account for tissue-specific differences. Finally, these in silico findings will require further validation through longitudinal studies to confirm the clinical utility of the identified biomarkers.

Overall, while this study provides important insights into the molecular mechanisms linking NSW to BC risk, our future research should focus on validating these biomarkers in diverse populations and exploring their role in early detection and prevention strategies. In particular, validation data at different timepoints in both saliva and/or oral mucosal cell samples from NSWs will be needed to establish a more precise temporal profile of gene and miRNA expression changes associated with circadian disruption. To facilitate the clinical application of our findings, future studies should prioritize the development of standardized protocols for the quantification of clock gene-related biomarkers in clinical and primary care settings. This includes determining optimal sampling times to account for circadian variability, utilizing non-invasive sampling techniques such as peripheral blood or saliva, and employing high-throughput, sensitive methodologies such as RT-qPCR and next-generation sequencing for the precise measurement of gene expression and miRNA profiles.

Such validation might pave the way for early detection strategies and personalized risk assessment in women at increased risk of BC due to prolonged exposure to NSW, with significant implications for occupational health, as it offers a new avenue for monitoring the health of employees exposed to circadian disruption, potentially leading to the implementation of personalized intervention strategies to mitigate BC risk. Once validated, these genetic and epigenetic biomarkers might be useful to enhance screening programmes, enabling more targeted prevention efforts. Integrating circadian biology into occupational health practises could lead to the development of useful guidelines that minimize circadian disruption, thereby reducing both the short- and long-term health effects linked with night shift work.

## 4. Materials and Methods

### 4.1. Dataset Repositories and Gene Expression Analyses

The Gene Expression Omnibus (GEO) database-deposited datasets considered in this study are described in Table 2. Regarding GSE115577, normalized gene expression data were obtained through the use of the R2 Genomics Analysis and Visualization Platform [39]. Meanwhile, the GEO2R Bioinformatic tool was employed for GSE122541, GSE107537, GSE164641, and GSE166044 [40]. For each dataset, differential gene expression analyses were performed between the control and experimental groups, and the results were expressed as the log_2_ fold change (log_2_FC). The estimated *p*-value was corrected for multiple testing by the Benjamini and Hochberg false discovery rate (FDR). FDR values ≤ 0.05 were considered statistically significant.

The tumour and normal breast single-cell RNA-Seq datasets analysed in this study are listed in Table 3. Differential gene expression analyses and multiple gene expression dot plots were generated by using the Broad Institute Single-Cell Portal interface [44].

The Single-Cell Portal calculates differential expression results using a Mann–Whitney U Test (or Wilcoxon signed-rank test) implemented with Scanpy’s rank_genes_groups function [48]. For each gene in a comparison, this method tests whether each normalized gene expression value is consistently higher (or lower) in one group of cells compared to another. The test output is a z-score, expressed as scaled mean expression. The FDR-adjusted *p*-value for each gene indicates whether the z-score is statistically significant (with values ≤ 0.05 considered statistically significant). The results are represented in the portal interface with dot plots, showing both the magnitude (scaled mean expression) and prevalence (proportion of cells expressing the gene) of gene expression.

### 4.2. Graphical Representations and Functional Analyses of Candidate Clock Genes and BC Genes

Gene enrichment analyses were carried out through the use of the g: Profiler online tool [49]. Furthermore, the STRING v11.0 bioinformatic tool was utilized to analyse the interaction networks of differentially expressed clock genes [50]. To identify miRNAs regulating the clock genes’ expression, an miRNA regulatory network analysis was performed by using the MirNet online tool (which integrates data from 14 miRNA databases: TarBase, miRTarBase, miRecords, miRanda, miR2Disease, HMDD, PhenomiR, SM2miR, PharmacomiR, EpimiR, starBase, TransmiR, ADmiRE, and TAM 2.0). To refine the outcome result, specificity filters were further applied (i.e., degree cut-off, betweenness centrality, shortest-path filter, minimum network) [51]. BC and melatonin pathway representations were obtained with the WikiPathways open-source biological pathway database [52]. Circadian clock pathway representation was generated by using the Reactome Knowledgebase data resource [53].

### 4.3. Statistical Analysis

Data processing and statistical analyses were performed using GraphPad Prism version 9.0 for Windows (GraphPad Software, La Jolla, CA, USA). The results were presented as mean ± standard deviation (SD). For each variable of interest, Shapiro–Wilk normality tests were run, and appropriate tests were used as described. Single-parameter comparisons between two groups were conducted using the two-tailed unpaired or paired Student’s *t*-test or Mann–Whitney U test or Wilcoxon signed-rank test. Single-parameter comparisons between three or more groups were performed using a one-way analysis of variance (ANOVA) or the Friedman test.

Correlations between different parameters were evaluated by calculating the Pearson’s or Spearman’s correlation coefficients. Correlograms were generated by using the SRplot free online visualization and graphic platform [54].

To assess the prognostic significance of deregulated clock genes, receiver operating characteristic curve (ROC) analyses were performed, and the corresponding areas under the curve (AUC) were calculated. Specifically, for each gene analysed, gene expression values were stratified into two classes (experiment vs. control), and subsequently, the two groups were analysed through the ROC function in Prism.

Recurrence-free survival (RFS) and distant recurrence-free survival (DRFS) analyses were conducted by using the Kaplan–Meier method in Prism, which accounts for censored data and provides a probabilistic estimate of survival over time for each group. Survival curves were compared using the log-rank test for trend, which assumes proportional hazards between groups. Median gene expression values were considered to be cut-off. For all the statistical analyses performed in this study, differences were considered significant with *p*-values < 0.05, with * *p* < 0.05; ** *p* < 0.01; *** *p* < 0.001; **** *p* < 0.0001.

## Figures and Tables

**Table 1:** Characteristics of the NHSI/NHSII cohorts.

	*n*	%
**Cohort**		
NHS I	318	51.04
NHS II	305	48.96
**Diagnosis year**		
Prior to 1990	11	1.77
1990–1999	304	48.80
2000–2011	308	49.44
**Age at the diagnosis**		
mean (SD)	56.8 (10.8)	
<50	173	27.77
50–59	210	33.71
60–69	141	22.63
>69	99	15.89
**IHC Subtype**		
Basal	59	9.47
HER2+	24	3.85
Luminal A	247	39.65
Luminal B	187	30.02
Missing	96	15.41
Unclassified	10	1.61
**Grade**		
G 1	146	23.43
G 2	305	48.96
G 3	144	23.11
n.a.	28	4.49
**Stage**		
S I	384	61.64
S II	185	29.70
S III	48	7.70
S IV	4	0.64
n.a.	2	0.32
**Surgery**		
None	1	0.16
Lumpectomy	278	44.62
Mastectomy	249	39.97
Unknown	95	15.25
**Treatment**		
None	19	3.05
Chemotherapy	41	6.58
Radiotherapy	25	4.01
Hormonal therapy	83	13.32
Mixed	379	60.83
Unknown	76	12.20
**Recurrence**		
yes	91	14.61
no	532	85.39

**Table 2:** List of Gene Expression Omnibus (GEO) databases analysed in the study.

GEO ID	Contributors	Platform	Normalization	Samples (*H. sapiens*)	Reference
GSE115577	Tamimi RM et al.	Affymetrix HTA 2.0	RMA	1246 (623 NA, 623 BC)	[26]
GSE122541	Gamble K et al.	Illumina HT-12 4.0	Custom	44 (22 DS, 22 NS)	[41]
GSE107537	Kervezee L et al.	Affymetrix HT Clariom S	RMA	103 (52 DO, 51 NO)	[42]
GSE164641	Marino N et al.	Illumina HiSeq 4000	DESeq2	162 (91 AV, 71 HI)	[24]
GSE166044	Marino N et al.	Illumina NextSeq 500	DESeq2	30 (15 HC, 15 SU)	[43]

Abbreviations. NA, normal adjacent; BC, breast cancer; DO, day-oriented; NO, night-oriented; DS, day shift; NS, night shift; AV, average; HI, high; HCs, healthy controls; SU, susceptible.

**Table 3:** Single-cell RNA-Seq datasets analysed in the study.

Study ID	Technology	Number of Cells	Type of Cells (*H. Sapiens*)	Reference
SCP1039	Illumina NextSeq 500	100,064	Surgically resected breast cancer tissue	[45]
SCP1106	Illumina NextSeq 500	24,271	Surgically resected breast cancer tissue	[46]
SCP1731	Illumina HiSeq X Ten	52,681	Surgically resected normal breast tissue	[47]

## Data Availability

The data generated in the present study may be requested from the corresponding author.

## Supplementary Materials

The following supporting information can be downloaded at https://www.mdpi.com/article/10.3390/ijms25168659/s1.
