# Supplementary material for: Computational Analyses Reveal Deregulated Clock Genes Associated with Breast Cancer Development in Night Shift Workers
Source: Int J Mol Sci. 2024 Aug 8;25(16):8659. doi: 10.3390/ijms25168659 (PMC11355052; doi:10.3390/ijms25168659)
Supplement: Supplementary file 1 [file ijms-25-08659-s001.zip › ijms-3131757-supplementary.pdf]

Supplementary Material

Computational analyses reveal deregulated clock genes associated with breast cancer development in night shift workers

Silvia Vivarelli [1,*], Giovanna Spatari [1], Chiara Costa [2], Federica Giambò [1] and Concettina Fenga [1]

[1] Department of Biomedical and Dental Sciences, Morphological and Functional Imaging, Section of Occupational Medicine, University of Messina, Messina, Italy

[2] Department of Clinical and Experimental Medicine, University of Messina, Messina, Italy

[*] Correspondence to:

Silvia Vivarelli, Department of Biomedical and Dental Sciences, Morphological and Functional Imaging, Section of Occupational Medicine, University of Messina, Via Consolare Valeria 1, 98124, Messina, Italy; silvia.vivarelli@unime.it

Table S1. Clock genes and BC genes analyzed in NHS dataset (GSE115577)

HGNC Symbol	Description	Gene ID	Probe ID
BHLHE40	basic helix-loop-helix family member e40	8553	TC03000015.hg
BHLHE41	basic helix-loop-helix family member e41	79365	TC12001324.hg
BMAL1	basic helix-loop-helix ARNT like 1	406	TC11000206.hg
BMAL2	basic helix-loop-helix ARNT like 2	56938	TC12000264.hg
CIART	circadian associated repressor of transcription	148523	TC01001183.hg
CLOCK	clock circadian regulator	9575	TC04002556.hg
CRY1	cryptochrome circadian regulator 1	1407	TC12001925.hg
CRY2	cryptochrome circadian regulator 2	1408	TC11000388.hg
DBP	D-box binding PAR bZIP transcription factor	1628	TC19001688.hg
HLF	HLF transcription factor, PAR bZIP family member	3131	TC17000693.hg
MTNR1A	melatonin receptor 1A	4543	TC04002950.hg
MTNR1B	melatonin receptor 1B	4544	TC11002828.hg
NFIL3	nuclear factor, interleukin 3 regulated	4783	TC09001325.hg
NPAS1	neuronal PAS domain protein 1	4861	TC19000671.hg
NPAS2	neuronal PAS domain protein 2	4862	TC02000611.hg
NPAS3	neuronal PAS domain protein 3	64067	TC14000212.hg
NPAS4	neuronal PAS domain protein 4	266743	TC11000667.hg
NR1D1	nuclear receptor subfamily 1 group D member 1	9572	TC17002615.hg
NR1D2	nuclear receptor subfamily 1 group D member 2	9975	TC03000127.hg
PDPK1	3-phosphoinositide dependent protein kinase 1	5170	TC16000787.hg
PER1	period circadian regulator 1	5187	TC17002849.hg
PER2	period circadian regulator 2	8864	TC02002914.hg
PER3	period circadian regulator 3	8863	TC01000100.hg
RORA	RAR related orphan receptor A	6095	TC15002563.hg
RORB	RAR related orphan receptor B	6096	TC09000338.hg
RORC	RAR related orphan receptor C	6097	TC01006373.hg
SIM1	SIM bHLH transcription factor 1	6492	TC06001969.hg
TEF	TEF transcription factor, PAR bZIP family member	7008	TC22000336.hg
TIMELESS	timeless circadian regulator	8914	TC12001605.hg
ERBB2	erb-b2 receptor tyrosine kinase 2	2064	TC17000480.hg
ERBB3	erb-b2 receptor tyrosine kinase 3	2065	TC12002907.hg
ERBB4	erb-b2 receptor tyrosine kinase 4	2066	TC02002735.hg
ESR1	estrogen receptor 1	2099	TC06003133.hg
ESR2	estrogen receptor 2	2100	TC14001211.hg
IL1A	interleukin 1 alpha	3552	TC02002218.hg
IL1B	interleukin 1 beta	3553	TC02002219.hg
IL6	interleukin 6	3569	TC07000137.hg
VDR	vitamin D receptor	7421	TC12002858.hg

Abbreviations: HGNC, HUGO Gene Nomenclature Committee.

Figure S1. Differential expression of the panel of 12 clock genes significantly deregulated in NHSI and NHSII (GSE115577, 623 paired samples). N = Normal adjacent breast samples; BC = Breast cancer samples. ** p < 0.01; *** p < 0.001; **** p < 0.0001.

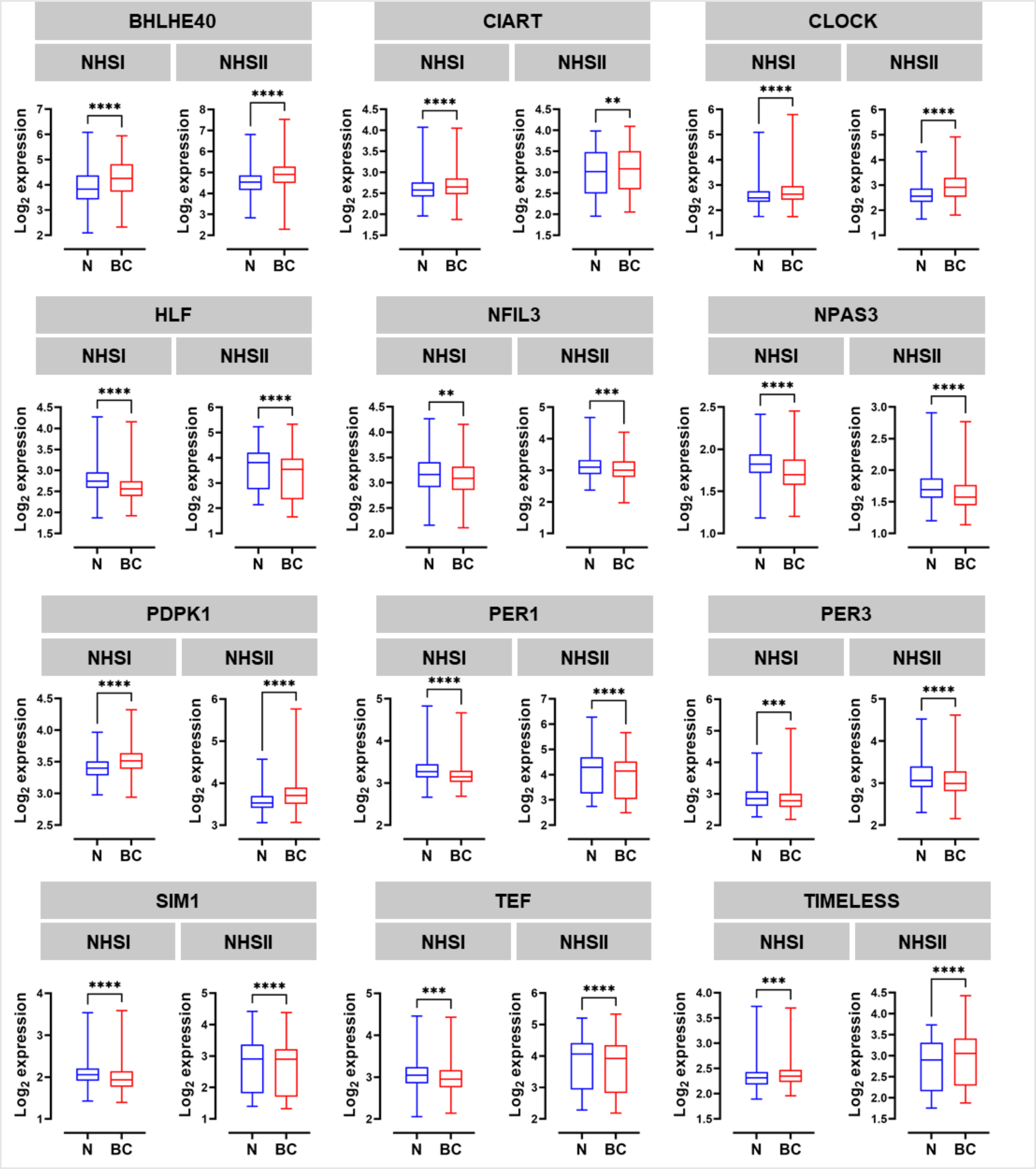

Figure S2. (A) Wiki-pathway: macrophage-stimulating protein (MSP) signaling network map (WP5353). Species: *H.sapiens*. (B) Wiki-pathway: clock-controlled autophagy in bone metabolism (WP5205). Species: *H.sapiens*.

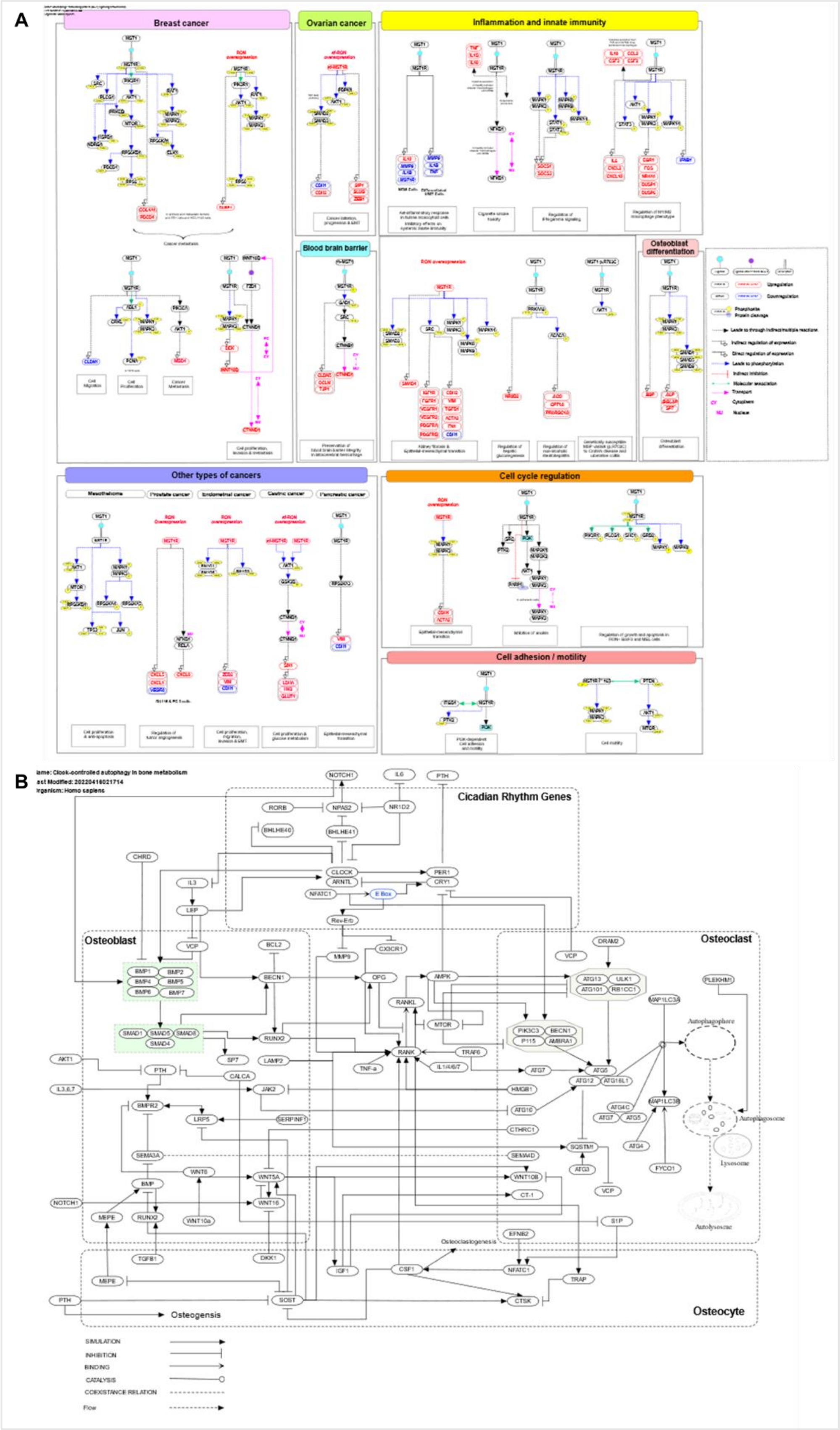

Figure S3. Stratification of significantly deregulated clock genes in GSE115577 based on tumor stage. Violin plots with median of 12 significantly deregulated clock genes in BC samples stratified based on their stage divided in Stage I and II (N=569) versus Stage III and IV (N=52). * p < 0.05; ** p < 0.01; *** p < 0.001; **** p < 0.0001, ns = not significant.

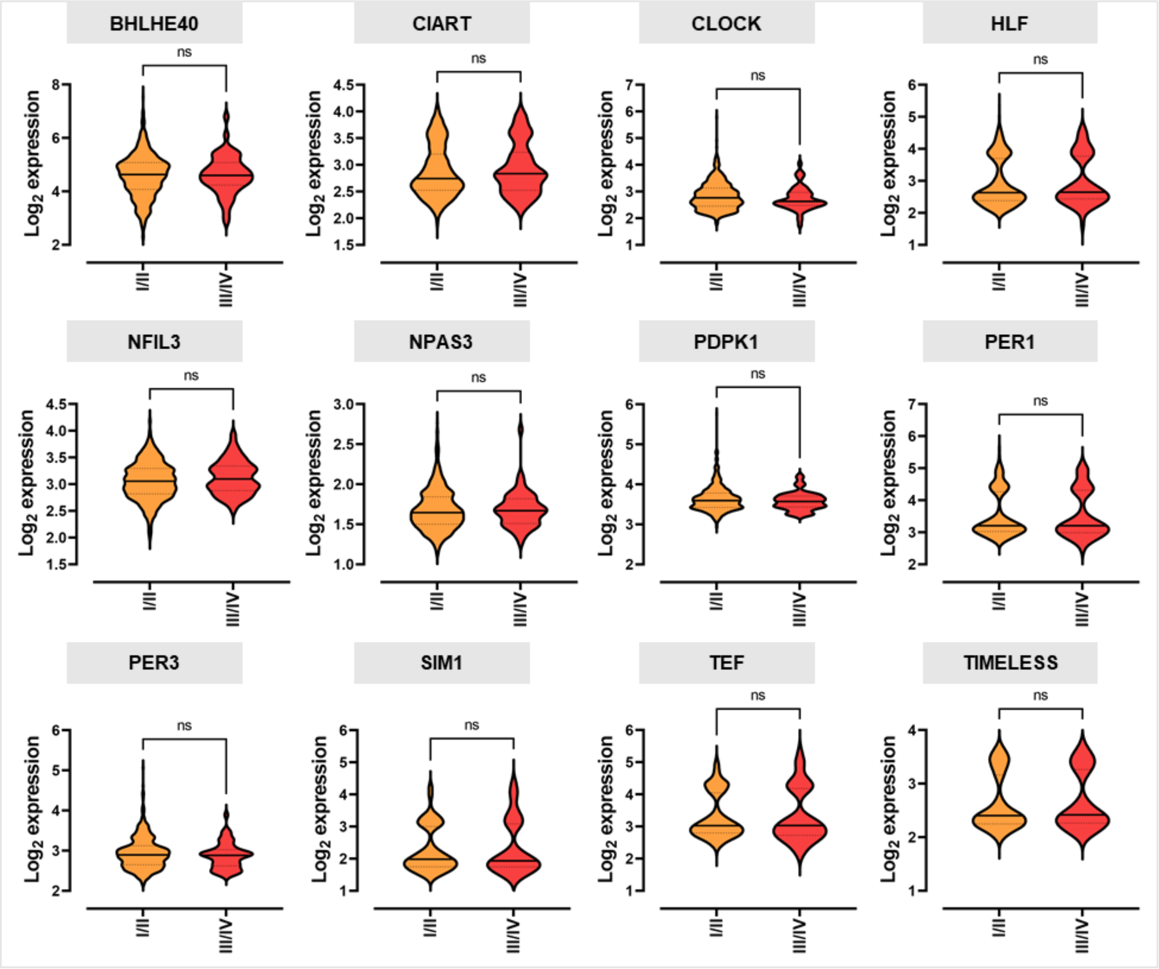

Figure S4. Kaplan–Meier analyses of 12 clock genes (GSE115577). A. Recurrence-free survival (RFS) correlated with clock genes expression (high:low = 50%:50%). B. Distant recurrence-free survival (DRFS) correlated with clock genes expression (high:low = 50%:50%). Survival curve comparison through Log-rank test; per each clock gene Chi square result and P value significance has been reported in light blue.

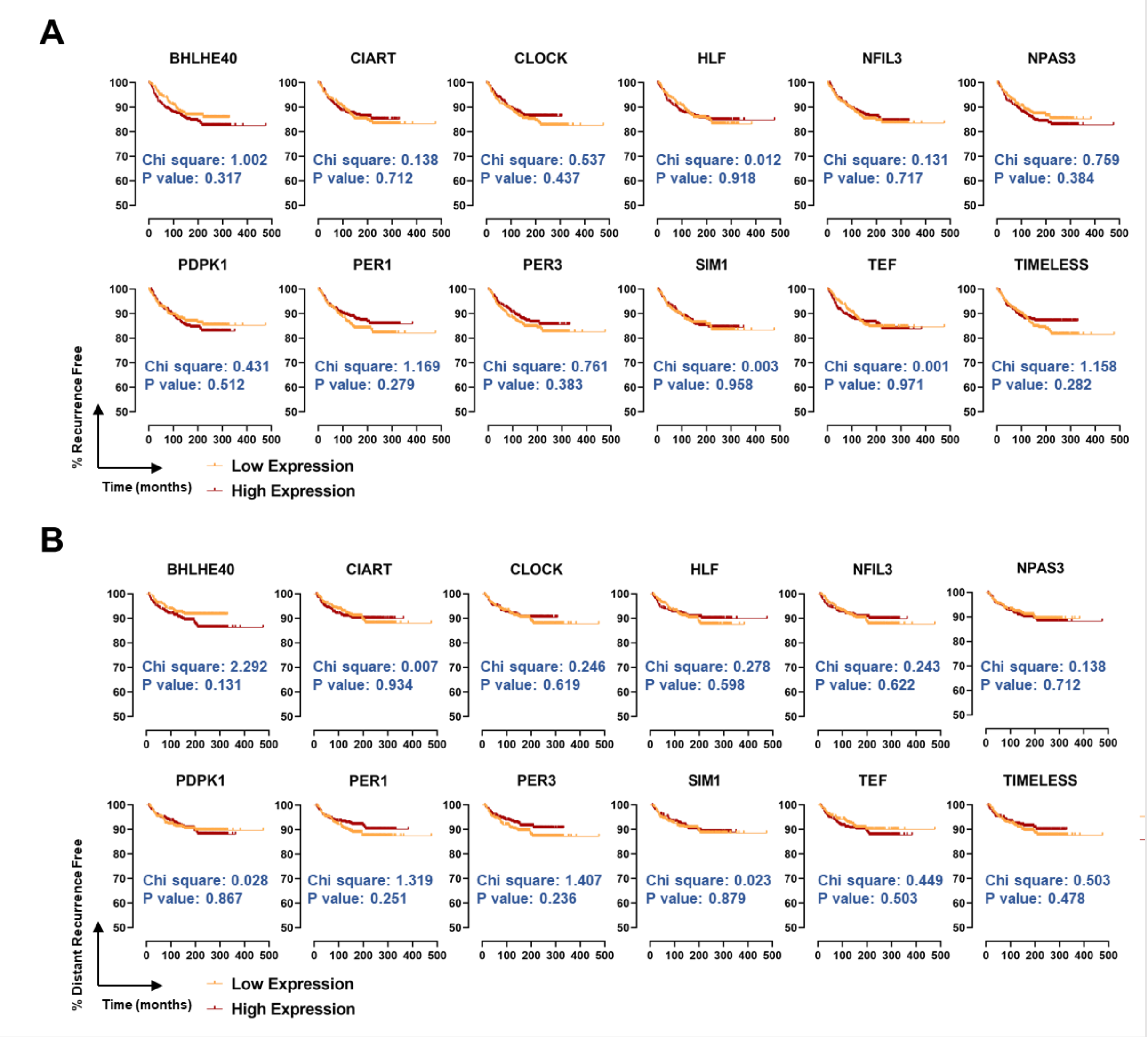

Figure S5. Clock genes expression during simulated night shift protocol. A. Rhythmic normalized expression (mean log2 expression ± SEM) of selected clock genes across time of day (in hours) in PBMCs from day-shift (N=3, orange line) and night-shift (N=3, light blue line) healthy nurses (GSE122541). B. Rhythmic normalized expression (mean log2 expression ± SEM) of selected clock genes across time of day (in relative hours) in PBMCs at baseline (N=8, grey line) versus night shift work (N=8, dark blue line, GSE107537). n.s.= not significant.

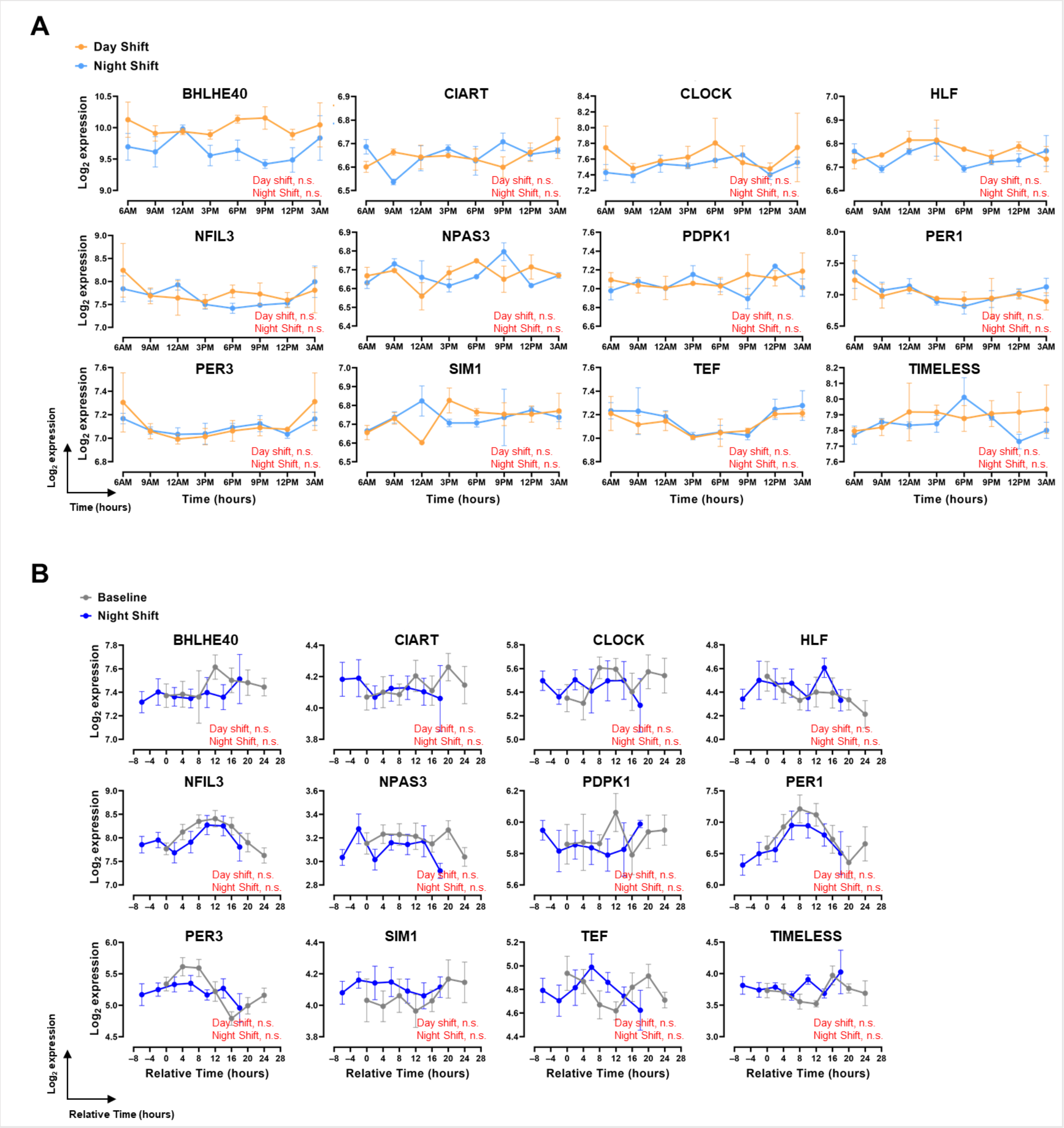

Table S2. G:profiler/g:GOSt functional analysis outcome for 12 clock genes in Gene Ontology (GO) pathways. Significant enrichment of: 15 GO molecular functions (GO:MF), 40 GO biological processes (GO:BP) and 5 GO cellular components (GO:CC).

source	term_name	term_id	adjusted_p_value	negative_log10_of_adjusted_p_value	intersection_size	intersections
GO:MF	transcription cis-regulatory region binding	GO:0000976	5,23E-08	7,281405369	10	BHLHE40,CIART,CLOCK,HLF,NFIL3,NPAS3,PER1,PER3,SIM1,TEF
GO:MF	transcription regulatory region nucleic acid binding	GO:0001067	5,30E-08	7,275606253	10	BHLHE40,CIART,CLOCK,HLF,NFIL3,NPAS3,PER1,PER3,SIM1,TEF
GO:MF	sequence-specific double-stranded DNA binding	GO:1990837	7,80E-08	7,108026559	10	BHLHE40,CIART,CLOCK,HLF,NFIL3,NPAS3,PER1,PER3,SIM1,TEF
GO:MF	double-stranded DNA binding	GO:0003690	1,42E-07	6,848856095	10	BHLHE40,CIART,CLOCK,HLF,NFIL3,NPAS3,PER1,PER3,SIM1,TEF
GO:MF	sequence-specific DNA binding	GO:0043565	1,57E-07	6,804581422	10	BHLHE40,CIART,CLOCK,HLF,NFIL3,NPAS3,PER1,PER3,SIM1,TEF
GO:MF	DNA binding	GO:0003677	2,62E-07	6,581223878	11	BHLHE40,CIART,CLOCK,HLF,NFIL3,NPAS3,PER1,PER3,SIM1,TEF,TIMELESS
GO:MF	RNA polymerase II transcription regulatory region sequence-specific DNA binding	GO:0000977	1,23E-06	5,909234627	9	BHLHE40,CIART,CLOCK,HLF,NFIL3,NPAS3,PER1,SIM1,TEF
GO:MF	E-box binding	GO:0070888	6,72E-06	5,172710317	4	BHLHE40,CIART,CLOCK,PER1
GO:MF	RNA polymerase II cis-regulatory region sequence-specific DNA binding	GO:0000978	2,93E-04	3,533453874	7	BHLHE40,CIART,CLOCK,HLF,NFIL3,PER1,TEF
GO:MF	cis-regulatory region sequence-specific DNA binding	GO:0000987	3,34E-04	3,47689189	7	BHLHE40,CIART,CLOCK,HLF,NFIL3,PER1,TEF
GO:MF	DNA-binding transcription factor activity, RNA polymerase II-specific	GO:0000981	7,28E-04	3,138001061	7	BHLHE40,CLOCK,HLF,NFIL3,NPAS3,SIM1,TEF
GO:MF	DNA-binding transcription factor activity	GO:0003700	1,11E-03	2,954515568	7	BHLHE40,CLOCK,HLF,NFIL3,NPAS3,SIM1,TEF
GO:MF	nucleic acid binding	GO:0003676	1,12E-03	2,949472109	11	BHLHE40,CIART,CLOCK,HLF,NFIL3,NPAS3,PER1,PER3,SIM1,TEF,TIMELESS
GO:MF	organic cyclic compound binding	GO:0097159	1,54E-03	2,812870223	12	BHLHE40,CIART,CLOCK,HLF,NFIL3,NPAS3,PDPK1,PER1,PER3,SIM1,TEF,TIMELESS
GO:MF	transcription regulator activity	GO:0140110	8,36E-03	2,077679513	7	BHLHE40,CLOCK,HLF,NFIL3,NPAS3,SIM1,TEF
GO:BP	rhythmic process	GO:0048511	6,01E-12	11,22114984	9	BHLHE40,CIART,CLOCK,HLF,NFIL3,PER1,PER3,TEF,TIMELESS
GO:BP	circadian rhythm	GO:0007623	7,96E-09	8,098847605	7	BHLHE40,CIART,CLOCK,NFIL3,PER1,PER3,TIMELESS
GO:BP	circadian regulation of gene expression	GO:0032922	3,82E-07	6,41846669	5	BHLHE40,CIART,CLOCK,PER1,PER3
GO:BP	photoperiodism	GO:0009648	1,47E-06	5,832596373	4	BHLHE40,CLOCK,PER1,PER3
GO:BP	regulation of circadian rhythm	GO:0042752	4,37E-06	5,359299213	5	BHLHE40,CLOCK,PER1,PER3,TIMELESS
GO:BP	regulation of DNA-templated transcription	GO:0006355	3,20E-05	4,495422857	11	BHLHE40,CIART,CLOCK,HLF,NFIL3,NPAS3,PER1,PER3,SIM1,TEF,TIMELESS
GO:BP	regulation of RNA biosynthetic process	GO:2001141	3,38E-05	4,47089921	11	BHLHE40,CIART,CLOCK,HLF,NFIL3,NPAS3,PER1,PER3,SIM1,TEF,TIMELESS
GO:BP	DNA-templated transcription	GO:0006351	4,67E-05	4,330388121	11	BHLHE40,CIART,CLOCK,HLF,NFIL3,NPAS3,PER1,PER3,SIM1,TEF,TIMELESS
GO:BP	RNA biosynthetic process	GO:0032774	5,15E-05	4,28840418	11	BHLHE40,CIART,CLOCK,HLF,NFIL3,NPAS3,PER1,PER3,SIM1,TEF,TIMELESS
GO:BP	regulation of RNA metabolic process	GO:0051252	8,29E-05	4,081548987	11	BHLHE40,CIART,CLOCK,HLF,NFIL3,NPAS3,PER1,PER3,SIM1,TEF,TIMELESS
GO:BP	regulation of nitrogen compound metabolic process	GO:0051171	1,23E-04	3,908374349	12	BHLHE40,CIART,CLOCK,HLF,NFIL3,NPAS3,PDPK1,PER1,PER3,SIM1,TEF,TIMELESS
GO:BP	regulation of primary metabolic process	GO:0080090	1,72E-04	3,765544736	12	BHLHE40,CIART,CLOCK,HLF,NFIL3,NPAS3,PDPK1,PER1,PER3,SIM1,TEF,TIMELESS
GO:BP	nucleobase-containing compound biosynthetic process	GO:0034654	1,72E-04	3,763230219	11	BHLHE40,CIART,CLOCK,HLF,NFIL3,NPAS3,PER1,PER3,SIM1,TEF,TIMELESS
GO:BP	regulation of nucleobase-containing compound metabolic process	GO:0019219	1,90E-04	3,721487815	11	BHLHE40,CIART,CLOCK,HLF,NFIL3,NPAS3,PER1,PER3,SIM1,TEF,TIMELESS
GO:BP	heterocycle biosynthetic process	GO:0018130	2,12E-04	3,673265085	11	BHLHE40,CIART,CLOCK,HLF,NFIL3,NPAS3,PER1,PER3,SIM1,TEF,TIMELESS
GO:BP	aromatic compound biosynthetic process	GO:0019438	2,18E-04	3,660718941	11	BHLHE40,CIART,CLOCK,HLF,NFIL3,NPAS3,PER1,PER3,SIM1,TEF,TIMELESS
GO:BP	organic cyclic compound biosynthetic process	GO:1901362	3,20E-04	3,495184749	11	BHLHE40,CIART,CLOCK,HLF,NFIL3,NPAS3,PER1,PER3,SIM1,TEF,TIMELESS
GO:BP	entrainment of circadian clock by photoperiod	GO:0043153	3,96E-04	3,401994415	3	BHLHE40,PER1,PER3
GO:BP	entrainment of circadian clock	GO:0009649	7,14E-04	3,146133802	3	BHLHE40,PER1,PER3
GO:BP	cellular nitrogen compound biosynthetic process	GO:0044271	1,24E-03	2,906669005	11	BHLHE40,CIART,CLOCK,HLF,NFIL3,NPAS3,PER1,PER3,SIM1,TEF,TIMELESS
GO:BP	regulation of transcription by RNA polymerase II	GO:0006357	1,28E-03	2,892095235	9	BHLHE40,CLOCK,HLF,NFIL3,NPAS3,PER1,PER3,SIM1,TEF
GO:BP	regulation of macromolecule metabolic process	GO:0060255	1,33E-03	2,875665223	12	BHLHE40,CIART,CLOCK,HLF,NFIL3,NPAS3,PDPK1,PER1,PER3,SIM1,TEF,TIMELESS

GO: BP	regulation of cellular metabolic process	GO:0031323	1,44E-03	2,842732821	12	BHLHE40,CIART,CLOCK,HLF,NFIL3,NPAS3,PDPK1,PER1,PER3,SIM1,TEF,TIMELESS
GO: BP	transcription by RNA polymerase II	GO:0006366	1,93E-03	2,715191802	9	BHLHE40,CLOCK,HLF,NFIL3,NPAS3,PER1,PER3,SIM1,TEF
GO: BP	regulation of metabolic process	GO:0019222	3,34E-03	2,476794624	12	BHLHE40,CIART,CLOCK,HLF,NFIL3,NPAS3,PDPK1,PER1,PER3,SIM1,TEF,TIMELESS
GO: BP	negative regulation of DNA-templated transcription	GO:0045892	3,36E-03	2,47427863	7	BHLHE40,CIART,CLOCK,NFIL3,PER1,PER3,TIMELESS
GO: BP	negative regulation of RNA biosynthetic process	GO:1902679	3,60E-03	2,443779477	7	BHLHE40,CIART,CLOCK,NFIL3,PER1,PER3,TIMELESS
GO: BP	regulation of gene expression	GO:0010468	5,18E-03	2,285755146	11	BHLHE40,CIART,CLOCK,HLF,NFIL3,NPAS3,PER1,PER3,SIM1,TEF,TIMELESS
GO: BP	negative regulation of glucocorticoid receptor signaling pathway	GO:2000323	6,21E-03	2,206917311	2	CLOCK,PER1
GO: BP	negative regulation of RNA metabolic process	GO:0051253	6,26E-03	2,203188879	7	BHLHE40,CIART,CLOCK,NFIL3,PER1,PER3,TIMELESS
GO: BP	regulation of macromolecule biosynthetic process	GO:0010556	6,62E-03	2,179466686	11	BHLHE40,CIART,CLOCK,HLF,NFIL3,NPAS3,PER1,PER3,SIM1,TEF,TIMELESS
GO: BP	negative regulation of nitrogen compound metabolic process	GO:0051172	6,66E-03	2,176808556	8	BHLHE40,CIART,CLOCK,NFIL3,PDPK1,PER1,PER3,TIMELESS
GO: BP	regulation of cellular biosynthetic process	GO:0031326	8,39E-03	2,076457125	11	BHLHE40,CIART,CLOCK,HLF,NFIL3,NPAS3,PER1,PER3,SIM1,TEF,TIMELESS
GO: BP	regulation of glucocorticoid receptor signaling pathway	GO:2000322	8,69E-03	2,060926976	2	CLOCK,PER1
GO: BP	regulation of biosynthetic process	GO:0009889	8,89E-03	2,050874049	11	BHLHE40,CIART,CLOCK,HLF,NFIL3,NPAS3,PER1,PER3,SIM1,TEF,TIMELESS
GO: BP	negative regulation of nucleobase-containing compound metabolic process	GO:0045934	1,08E-02	1,965457526	7	BHLHE40,CIART,CLOCK,NFIL3,PER1,PER3,TIMELESS
GO: BP	response to abiotic stimulus	GO:0009628	2,28E-02	1,642141286	6	BHLHE40,CLOCK,PDPK1,PER1,PER3,TIMELESS
GO: BP	response to light stimulus	GO:0009416	3,17E-02	1,499611013	4	BHLHE40,CLOCK,PER1,PER3
GO: BP	glucocorticoid receptor signaling pathway	GO:0042921	3,22E-02	1,491877861	2	CLOCK,PER1
GO: BP	corticosteroid receptor signaling pathway	GO:0031958	3,76E-02	1,425068768	2	CLOCK,PER1
GO: CC	chromatin	GO:0000785	9,20E-06	5,036438165	8	BHLHE40,CLOCK,HLF,NFIL3,NPAS3,SIM1,TEF,TIMELESS
GO: CC	protein-DNA complex	GO:0032993	1,34E-05	4,872493437	8	BHLHE40,CLOCK,HLF,NFIL3,NPAS3,SIM1,TEF,TIMELESS
GO: CC	chromosome	GO:0005694	1,42E-04	3,847358164	8	BHLHE40,CLOCK,HLF,NFIL3,NPAS3,SIM1,TEF,TIMELESS
GO: CC	nucleus	GO:0005634	8,83E-03	2,054166805	12	BHLHE40,CIART,CLOCK,HLF,NFIL3,NPAS3,PDPK1,PER1,PER3,SIM1,TEF,TIMELESS
GO: CC	RNA polymerase II transcription regulator complex	GO:0090575	3,52E-02	1,453673301	3	CLOCK,HLF,NFIL3
